# Advances in Research on Marine-Derived Lipid-Lowering Active Substances and Their Molecular Mechanisms

**DOI:** 10.3390/nu15245118

**Published:** 2023-12-15

**Authors:** Lina Liu, Yihui Chen, Bei Chen, Min Xu, Shuji Liu, Yongchang Su, Kun Qiao, Zhiyu Liu

**Affiliations:** 1 College of Food Science, Fujian Agriculture and Forestry University, Fuzhou 350002, China; 3210910034@fafu.edu.cn (L.L.); harris2197395@163.com (Y.C.); 2 Engineering Research Center of Fujian and Taiwan Characteristic Marine Food Processing and Nutrition and Health, Ministry of Education, Fuzhou 350002, China; 3 Fisheries Research Institute of Fujian, Key Laboratory of Cultivation and High-Value Utilization of Marine Organisms in Fujian Province, Xiamen 361013, China; 13950001893@163.com (B.C.); xumin@jmu.edu.cn (M.X.); cute506636@163.com (S.L.); suyongchang@stu.hqu.edu.cn (Y.S.)

**Keywords:** marine bioactive substances, hyperlipidemia, lipid metabolism, molecular mechanisms

## Abstract

Hyperlipidemia (HLP) is a metabolic disorder caused by abnormal lipid metabolism. Recently, the prevalence of HLP caused by poor dietary habits in the population has been increasing year by year. In addition, lipid-lowering drugs currently in clinical use have shown significant improvement in blood lipid levels, but are accompanied by certain side effects. However, bioactive marine substances have been shown to possess a variety of physiological activities such as hypoglycemic, antioxidant, antithrombotic and effects on blood pressure. Therefore, the hypolipidemic efficacy of marine bioactive substances with complex and diverse structures has also attracted attention. This paper focuses on the therapeutic role of marine-derived polysaccharides, unsaturated fatty acids, and bioactive peptides in HLP, and briefly discusses the main mechanisms by which these substances exert their hypolipidemic activity in vivo.

## 1. Introduction

Cardiovascular disease (CVD) is a major cause of morbidity and mortality worldwide [1]. Among the types of non-communicable diseases, Cardiovascular disease (CVD) is the leading cause of morbidity and mortality, affecting over 523 million people globally [2]. Dyslipidemia and hypertension are the main risk factors for CVD [3]. Hyperlipidemia is a systemic metabolic abnormality caused by genetically or environmentally induced increases in plasma levels of cholesterol, triglycerides, and low-density lipoproteins and decreases in high-density lipoproteins. The current treatment for HLP consists of both drug therapy and dietary intervention. However, the lipid-lowering statin drugs have been shown to cause side effects in some individuals [4,5,6]. Therefore, dietary therapy is a preferred means of preventing HLP. Diet plays a role in both the prevention and treatment of HLP [7].

**Figure 1:**
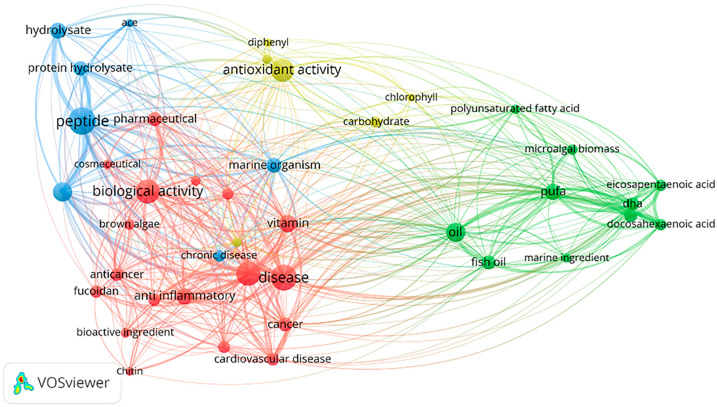
Research on the relationship between marine active substances and human health. The image was generated by software called VOSviewer 1.6.19 that analyzes search results from the web of science core dataset for the keywords (marine, bioactive compound). We extracted the keywords of the articles extremely references in the dataset and plotted them as images on this basis. Different bubble colors in the picture represent different categories, with the more relevant literature representing larger bubbles for the subject terms.

**Figure 2:**
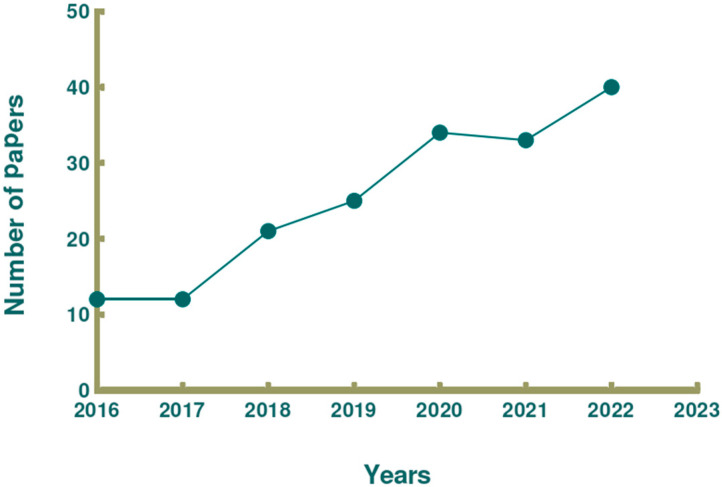
Number of publications on marine hypolipidemic active substances (2016–2023). We counted the number of articles per year related to the subject terms in web of science with keywords (marine, hyperlipemia, bioactive compound) and year of publication (2016–2023) constraints and made a line graph with the GraphPad Prism 8.0.

The oceans, which cover approximately 71% of the surface area of the Earth, are rich in biological resources, and the development of marine exploration technology and biotechnology has advanced considerably. Consequently, some countries with more marine resources have observed an increase in their production value in agriculture and animal husbandry, and the development of marine active substances has progressed considerably [8]. Changes in environmental factors such as the unique temperature, pressure, and light of the ocean create a diversity of marine organisms, which results in the production of a wide range of biologically active substances [9,10]. These substances include polyunsaturated fatty acids, proteins, pigments, vitamins, and minerals, which have been widely used as ingredients in functional foods [9,10]. According to the Web of Science core database, numerous scholars have started to focus on the relationship between marine active substances and human health over the past 10 years [11,12,13]. Furthermore, exploration of the extraction, purification, activity, and molecular mechanisms of action of polysaccharides, fish oils, proteins and active peptides derived from oceanic has now revealed the anticancer, lipid-lowering, and anti-inflammatory effects of some edible marine organisms [11,12,13]. Since 2016, the number of publications about marine-derived hypolipidemic active substances has been increasing. In this paper, we reviewed the types of discovered marine-derived hypolipidemic active substances and summarized the methods for evaluating their hypolipidemic efficacy and mechanism of action. Figure 1 shows a schematic illustration of how current research on marine active substances has impacted human health, and Figure 2 shows the number of publications on marine hypolipidemic substances over the period of a decade between 2016 and 2023.

## 2. Current Status of Research on Hyperlipidemia

**Figure 3:**
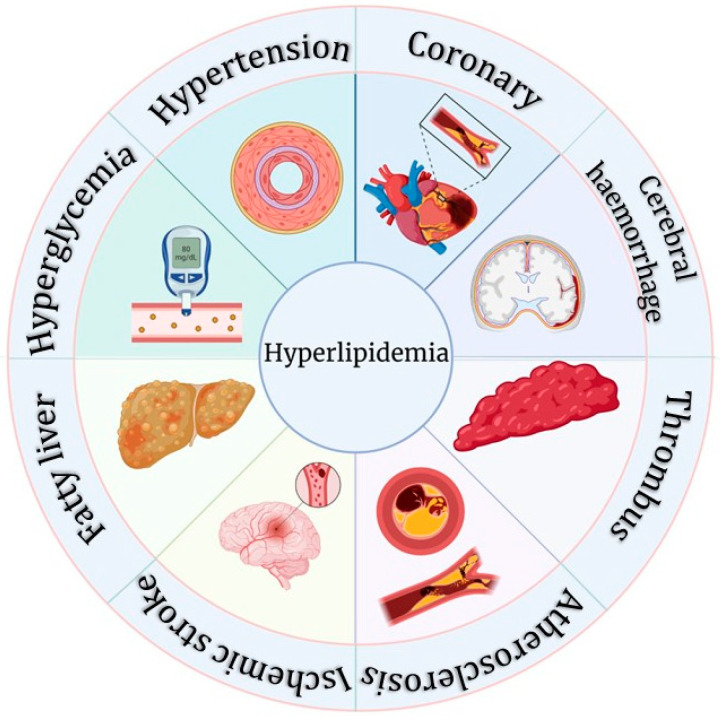
Complications of hyperlipidemia (HLP).

The continuous societal and the economic development and accompanied change in lifestyle of people has caused unhealthy dietary habits to gradually become the main factor leading to abnormal lipid metabolism. Elevated total cholesterol (TC), total triglycerides (TG), low-density lipoprotein (LDL), very low-density lipoprotein (VLDL), and phospholipids, as well as lower levels of high-density lipoprotein (HDL) in the blood are associated with alterations in atherosclerotic, cardiac, and coronary indices [14]. Excessive lipid levels in the blood can block blood vessels, which in turn can cause a variety of diseases such as fatty liver, atherosclerosis, and CVD, increasing the risk of hypertension, Alzheimer’s disease, pancreatitis, periodontitis, and hepatitis [15,16]. Therefore, treating HLP has a significantly positive impact on reducing the incidence of atherosclerosis, CVD, and cerebrovascular diseases. Figure 3 is a diagrammatic illustration of some health complications associated with HLP.

**Figure 4:**
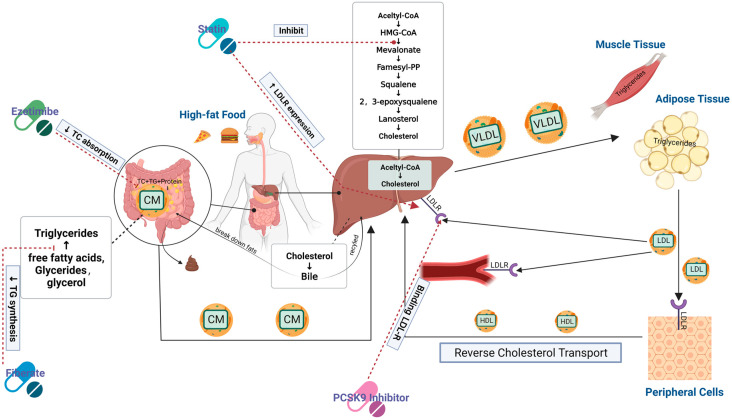
Lipid metabolism pathway and target of hypolipidemic drugs. This diagram mainly shows the main process mechanisms of cholesterol absorption, transport and metabolism in the human body, including endogenous and exogenous cholesterol metabolism. Exogenous cholesterol mainly comes from diet and is absorbed through the small intestine, while endogenous cholesterol is mainly synthesized by the body itself, mainly in the liver. In addition, it contains the location of the target sites of common lipid-lowering drugs.

Currently, the common clinical drugs used to treat HLP are statins, PCSK9 inhibitors, niacin, ezetimibe, probukau and betablockers. Although these drugs can reduce blood lipids levels in patients with HLP, they have certain side effects. The most important side effect of statins is elevation of creatine kinase (CK), myalgia, and rhabdomyolysis [17]. Although niacin is effective in lowering serum TC levels and increasing HDL cholesterol (HDL-C) levels [18], it has adverse effects including flushing, nausea, gastrointestinal distress and hepatotoxicity [19]. In light of this, there is also a trend towards drug combinations. For example, combination therapy with ezetimibe and statins can further reduce LDL-C, and it is recommended for situations where LDL-C targets cannot be achieved using maximal or maximally tolerated statin monotherapy regimens. Their combination can further reduce ASCVD risk without raising significant safety concerns, making it an effective treatment option [20]. Marine-derived active substances have become new drug lead molecules because of their structural diversity and complexity. At the same time, some studies have found that marine-derived bioactive materials are safe and effective, with novel mechanisms of action. Consequently, an increasing number of scholars are now working on the development of natural and non-toxic hypolipidemic active substances. Figure 4 shows some identified pathways for lipid metabolism and targets of potential hypolipidemic agents.

## 3. Source Classification of Marine-Derived Hypolipidemic Actives

**Figure 5:**
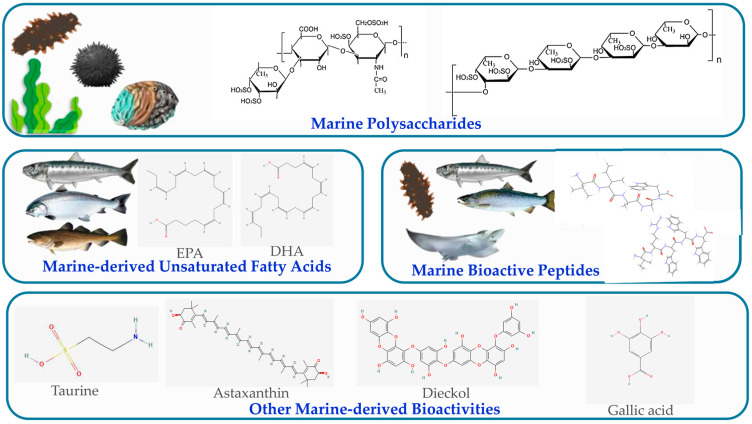
Marine-derived hypolipidemic actives.

A number of marine-derived bioactive compounds have been found to be involved in a wide range of biological processes, including activation of signal transduction pathways, antioxidant defense, protein expression, and the maintenance of mitochondrial integrity [21,22,23]. With the rapid development of extreme marine biotechnology, people are constantly discovering new compounds with medicinal value, and a large number of anti-tumor, anti-bacterial, anti-virus, anti-coagulant, antihypertensive and hypolipidemic bioactive substances can be extracted from the body of marine organisms in extreme environments [24]. Polysaccharides derived from seaweed, protein peptides obtained from the skin and skeleton of deep-sea fish, and polyunsaturated fatty acids rich in deep-sea fish oil have all been shown to have a variety of biological activities [25], for example, the antioxidant effect of polysaccharides and peptides, the cardiovascular-protective effect of fish oil, and the therapeutic effect of polysaccharides and protein peptides on hyperlipemia (Figure 5) [26,27,28]. We list in Table 1 the sources of marine-derived hypolipidemic actives and the methods of efficacy evaluation.

### 3.1. Marine Polysaccharides

Polysaccharides are divided into land-and sea-based types, which are mainly derived from marine organisms that live in a saline buffer system with a specific water pressure, high salinity, low temperature, insufficient light source, and low dissolved oxygen. These unique environmental factors lead to differences in synthesis pathways between land- and sea-derived substances, and marine polysaccharides have a novel structure and special biochemical mechanism because of the beneficial environmental factors [29]. According to their sources, marine polysaccharides can be categorized as algal (classified as brown, red, and green algae according to pigment deposition), marine animal, and marine microbial polysaccharides. Algal polysaccharides are the main components of macroalgae and phytoplankton organisms [30]. 

Furthermore, polysaccharides originating from algae have a complex structure, with highly polymerized branched chains and numerous reactive groups, which makes them show great potential bioactivity [31]. Most marine animal polysaccharides are found in connective tissues such as the body wall of marine invertebrates such as echinoderms (sea urchins and sea cucumbers), including sulfated polysaccharides and fucoidan in sea cucumbers [32]. The viscera of some marine animals are also a source of bioactive polysaccharides, such as sulfated polysaccharides extracted from abalone viscera with anticoagulant and hypolipidemic activities, and polysaccharides extracted from squid viscera with immunological activities [33,34,35]. 

Although animals and plants in the ocean can also produce polysaccharides, marine microorganisms are more popular sources because of their rapid reproductive rate and production of abundant and easily isolated polysaccharides [36]. Microorganisms are widely distributed on the seafloor and are highly adaptable to the environment. Furthermore, intracellular polysaccharides are mainly derived from the cell walls of marine fungi, bacteria, and actinomycetes. The secondary metabolites produced by microorganisms are referred to as extracellular polysaccharides, and include those produced by *Aspergillus versicolor N2bc* from the deep-sea fungus, which have antioxidant activity [37].

The development of purification and identification techniques has contributed to the gradual elucidation of the composition of polysaccharides, and their bioactivity has been shown to be closely related to their monosaccharide composition, the type of glycosidic bond, the number of hydroxyl groups, and the conformation of the polysaccharide chain [38,39]. Furthermore, natural polysaccharides with numerous glycoalkaloid acids are usually considered to have superior bioactivity [38,39]. Sulfated polysaccharides are able to interact with some biomolecules because of their sulfate functional groups and positive charges. Subsequent research led to the extraction of four types of sulfated polysaccharides from sea cucumbers (*Pearsonothuria graeffei* and *Isostichopus badionotus*) and investigated the relationship between structure and function [40]. This study finally found that all four sulfated polysaccharides exhibited hypolipidemic effects in obese rats, and those with a stretchy linear conformation displayed a more pronounced activity [40].

A marine-derived chitosan-oligosaccharide intervention significantly reduced plasma TC and increased HDL-C levels in hypercholesterolemic hamsters, and increased the relative abundance of *Bacteroidetes* in the intestine [41]. Wan et al. [42] found that polysaccharides from *Chlorella pyrenoidosa* (CPP) improved plasma and liver lipid metabolism and accelerated cecum total bile acid, short-chain fatty acid, and lipid metabolism. Furthermore, CPP also upregulated adenosine-monophosphate-activated protein kinase α (AMPKα) and downregulated the expression of acetyl-coenzyme A carboxylase, sterol regulatory element-binding protein 1c, and β-hydroxy-β-methylglutaryl coenzyme A [42]. 

In addition, *Sargassum pallidum* polysaccharides also improve hepatic lipid levels in the serum of mice induced by a high-fat diet (HFD) and significantly reduced fat accumulation in the liver and downregulated the expression levels of genes related to fat synthesis (FAS, SREBP-1c, and ACC) [43]. Sea cucumber has shown outstanding activity in study of active substances [39]. Liu et al. [39] used *Apostichoru japonicus* as raw material to prepare sea cucumber polysaccharide (AJP) using protease hydrolysis. AJP is mainly composed of aminoglucose, galactosamine, glucuronic acid, mannose, glucose, galactose, and fucose, with an average molecular weight of 36.2 kDa. 

Studies revealed that treatment of hyperlipidemic Wistar rats with AJP significantly reduced their serum TC, TG, and LDL-C levels, whereas the HDL-C level was significantly increased. Liu et al. [35] used a high voltage pulsed electric field to extract crude polysaccharides from *Haliotis discus hannai* viscera and infrared spectroscopy analysis revealed the presence of sugar and sulfate groups. Consequently, abalone visceral polysaccharides were shown to reduce TC, TG, and LDL-C levels, while increasing HDL-C levels in the plasma of mice exposed to a HFD [35]. Furthermore, the malondialdehyde (MDA) content decreased and superoxide dismutase (SOD) activity increased significantly [40].

The lipid-lowering activity of marine-derived polysaccharides is highlighted by sulfated polysaccharides, which are mostly derived from seaweeds and other marine plants. However, few studies have investigated the lipid-lowering effects of marine microbial polysaccharides. In addition, the hypolipidemic activity of polysaccharides is related to their structures. Therefore, the structural characterization of polysaccharides using chromatographic and spectroscopic techniques is useful for the screening of active compounds and studying of their hypolipidemic molecular mechanisms.

### 3.2. Marine-Derived Unsaturated Fatty Acids 

A typical feature of marine foods is that they are rich in docosahexaenoic acid (DHA) and eicosapentaenoic acid (EPA). Furthermore, dietary fatty acids from deep-sea fish such as salmon, cod, sardines, and Antarctic krill organisms have attracted much attention for their human health benefits [28,35,43,44,45,46,47]. Deep-sea fish oils are rich in unsaturated fatty acids, including the Omega-3 family of fatty acids, represented by EPA and DHA, which have received much attention because of their nutritional health functions [48]. Dietary Omega-3 polyunsaturated fatty acids in deep-sea fish oil supplementation improves hepatic lipid metabolism by regulating bile acid metabolism [49]. Moreover, DHA is a natural endogenous ligand for peroxisome proliferator-activated receptors (PPARs), which it activates to enhance fatty acid β-oxidation in the mitochondria [50,51,52]. This effect increases fatty acid catabolism, which in turn reduces plasma TG levels and, thus, consumption of EPA and DHA from deep-sea fish oils reduces the CVD-associated mortality rate [50,51,52]. Current clinical guidelines recommend a combination of DHA and EPA for the treatment of severe hypertriglyceridemia [53,54,55].

DHA activates AMPK, thereby inhibiting endoplasmic reticulum (ER) stress in the mitochondria [21]. DHA supplementation in the grass carp diet was also significantly reduced hepatic TG, MDA, serum tumor necrosis factor-α (TNFα), and nuclear transcription factor κB (NFκB) levels [21]. This effect inhibited palmitic acid (PA)-induced ER stress and lipid accumulation in vitro and *Ctenopharyngodon idella* hepatocyte inflammation [21]. Sabarinathan et al. [56] evaluated the protective effects of DHA- and EPA-rich fish oil against atherosclerosis using a high cholesterol diet-induced zebrafish model. There results showed that the fish oil-fed zebrafish group accumulated 40% less cholesterol than the regular diet group did [56]. 

In addition, compared to the normal diet fed group, the fatty acid synthesis (FAS) gene expression level in the livers of the fish oil fed group was significantly lower (*p* < 0.05) [56]. Oral DHA may have unstable bioavailability and, therefore, Zhang et al. [52] prepared a DHA liposome formulation as an injectable nanomedicine to avoid DHA degradation. This study found that DHA liposomes were easily phagocytosed by activated macrophages, exerted effective anti-inflammatory and antioxidant effects, and inhibited the formation of foam cells, further slowing down atherosclerosis development [52]. Tian et al. [22] isolated EPA-rich phosphatidylcholine (EPA-PC) and EPA-phosphatidylethanolamine (PE) from sea cucumber, and found that they both activated the transcription of PPARα/PPARγ. In addition, both compounds upregulated the expression of the target gene of lipid metabolism of PPARγ by dual-luciferase reporter in 3T3-L1 cells and inhibit the phosphorylation of PPARγ at Ser273. These effects, in turn, improved insulin resistance and abnormal lipid accumulation induced by a high-fat high-sucrose diet (HFSD) [22]. Krill oil also contains DHA and EPA, which when bound to phospholipids may have a higher bioavailability and absorption than that of exogenous omega-3 polyunsaturated fatty acids from fish oil and, thus, krill oil may have greater potential for the treatment of metabolic syndrome than fish oil does [57,58]. 

Krill oil supplementation decreased total TC, TG, and LDL-C levels in the liver and serum of hypercholesterolemic rats, as well as HMGCR activity [59]. Furthermore, krill oil increased TC and bile acid levels in the feces of experimental rats, and promoted bile acid metabolism and cholesterol efflux [59]. Liang et al. [60] found that a combination intervention using krill oil and *Bifidobacterium animalis* subsp. *Lactobacillus* F1-7 significantly reduced the atherosclerotic plaque area, anti-inflammatory factor levels and modulated the cholesterol 7-alpha hydroxylase (CYP7A1) pathway to reduce lipid accumulation in mice.

The global abundance of marine biological resources provides an important guaranteed source of material for the development and application of unsaturated fatty acids [61]. Currently, polyunsaturated fatty acids from deep-sea fish and shrimp have been developed into health products and medicines [62]. Furthermore, although the fishy odor limits its scope of application, recent advances in technology such as microencapsulation, fish oil water-in-emulsions, and other delivery systems have emerged as potential solutions [63]. These technologies not only remove the fishy odor, but they also improve the stability of the fish oil and bioavailability of active constituents. In addition, the advancement of such technologies to develop formulations for the application of fish oil in human health has created more possibilities and widened the scope of application of fish oil in human health management.

### 3.3. Marine Bioactive Peptides

Marine bioactive peptides have anti-inflammatory, antioxidant, anti-thrombotic, and other activities and have become a major research hotspot owing to their multiple bioactive functions, safety, and no toxicities [64]. Marine bioactive peptide is a general term for different short peptides with complex linear and cyclic structures consisting of 2–16 amino acid residues in different combinations and arrangements obtained from marine organisms such as fish, sponges, sea squirts, seaweeds and mollusks [25]. Conventional sources of marine bioactive peptides are generally the muscle or viscera, skin, shell, bone, and other by-products of marine organisms.

These substances are extracted using enzymes/hydrolysis (such as pepsin, trypsin, and alkaline protease), fermentation, acid-base extraction, and heat extraction followed by ultrafiltration and purification. The extracts are then analyzed to identify the components using chromatography in combination with the appropriate detector, to screen out the peptides with a high level of improved bioactivity. The activity of bioactive peptide is related to the protease used for extraction, the degree of hydrolysis, and the molecular weight of the product. Different proteases can cleave different sites, and the amino acid composition of the product will lead to different biological functions.

Furthermore, different degrees of hydrolysis generate peptides of different lengths, and a hydrolyzed product is more easily absorbed than a single amino acid or unhydrolyzed protein is. Recently, some researchers are of the opinion that shorter peptides with smaller molecular weight are more easily absorbed and more active [65,66]. Previously, peptides with hypolipidemic activity were obtained from marine organisms such as *Rhopilema esculentum* [67], *Corbicula fluminea Muller* [68], and *Sardinella aurita* [69]. Subsequently, an increasing number of researchers began to focus on developing bioactive peptides from marine organisms.

TetraSOD is a unique marine health and functional ingredient derived from the marine microalgae Tetraselmis chuii strain CCFM03, which has a history of use in food and nutraceutical applications around the world [70] TetraSOD promotes endogenous antioxidant defense mechanisms in the liver and modulates plasma markers of oxidative stress and inflammation, thereby ameliorating these effects associated with metabolic syndrome (MetS) [71]. Collagen from the skin of *Sphyrna mokarran* downregulates the expression of fatty acid synthase (FAS) and 3-hydroxy-3-methylglutaryl monoacyl-coenzyme A reductase (HMGCR), and upregulates the expression of lecithin-cholesterol acyltransferase (LCAT) in the liver to alleviate cholesterol accumulation [72]. Wergedahl et al. [73] used lipid-free enzyme digest (a mixture of small peptides and free amino acids) prepared using enzymatic hydrolysis of *Salmo salar*, L. with Protamex^TM^ protease as a diet and found that it increased the HDL-C/TC ratio in rat plasma. Furthermore, the process further lowered rat plasma cholesterol by decreasing the activity of hepatic HMGCR in Zucker (fa/fa) obese rats [73]. Fish skin collagen peptides also have the ability to enhance cholesterol metabolism, and *Sphyrna mokarran* skin collagen peptides can alleviate HLP induced by a HFD with alcohol [72].

Lee et al. [74] investigate the effects of enzymatically obtained tuna skin collagen peptides from 3T3-L1 preadipocytes in a HFD-induced obese mouse model, and found that subcritical hydrolysis of fish collagen peptide significantly inhibited lipid accumulation during 3T3-L1 cell differentiation. In addition, this substance also significantly reduced serum TC, TG, and LDL levels, whereas it increased serum HDL levels in the obese mice [74]. Collagen peptides from the skin of *Raja kenojei* have also demonstrated an anti-obesity effect on lipid metabolism in mice fed a HFD [25].

Furthermore, collagen peptides significantly inhibited lipid accumulation and increased serum HDL levels in obese mice through downregulation of fatty acid synthesis (sterol regulatory element-binding protein-1 (SREBP-1), FAS, and acetyl coenzyme A carboxylase (ACC), sterol regulatory element-binding protein 2 (SREBP-1), SREBP-2, and HMGCR in the liver of mice [23]. It also upregulated the expression levels of proteins used for β-oxidation (PPAR-α and carnitine palmitoyltransferase 1 (CPT1) to inhibit fat accumulation [23]. Recently, to better define the molecular mechanism of bioactive proteins, studies have used purification methods such as chromatography to identify the peptide sequences in the active proteins [75].

Glycine (Gly) was abundant in the enzymatic digest of *Zosterissessor ophiocephalus* under the action of alkaline protease, which reduced the enzyme activity of serum HMGCR [46]. Furthermore, this effect downregulated the expression of the LDL receptor (LDLR) in HFD rats, resulting in a blockade of cholesterol synthesis [46]. The hypolipidemic activity of two pentapeptides (VIAPW and IRWWW) identified from the muscle digests of *Miichthys miiuy* on oleic acid (OA)-induced lipid accumulation in HepG2 cells, was exhibited by their significant dose-dependent inhibition of OA-induced lipid accumulation and reduction of intracellular levels of TG and TC [76]. Both pentapeptides downregulated the expression levels of *SREBP-1c*, *SREBP-2*, *FAS*, *ACC*, and *HMGCR* genes in lipid synthesis and upregulated the expression levels of *PPARα*, *ACOX-1*, and *CPT-1* genes in lipid oxidation [76].

With the existence of hundreds of peptides, rapidly screen out target peptides using traditional evaluation methods is difficult and, therefore, computer-aided drug design plays an important role in facilitating the process. Target identification is the first step in modern drug development, because most important physiological processes in organisms, such as cell cycle regulation, anabolism, signal transduction, and transmission of genetic information are closely dependent on the identification of proteins and ligands and their interactions. Some computer software such as Discovery studio, Autodock, Pymol, and MOE can predict the affinity and binding stability of both [77].

This is achieved by analyzing the intermolecular electrostatic interactions between the active molecule and the receptor amino acid residues, and the effects of interactive forces such as ionic bonding, hydrogen bonding, and van der Waals’ forces. Zhao et al. [78] used ultrafiltration and molecular exclusion chromatography to isolate *Ostrea rivularis Gould* protein, which was followed by purification using ultrafiltration and molecular exclusion chromatography [78]. Subsequently, three novel peptides with strong xanthine oxidase (XO) inhibitory activity (ALSGSW, GGYGIF, and MAIGLW) were screened using liquid chromatography-tandem mass spectrometry (LC-MS/MS) identification and molecular docking techniques [78].

Furthermore, the mechanism of the peptide-XO interaction was revealed using molecular docking techniques, the structures of the peptides were rationally designed based on this information, and the results showed that replacing the GGYGIF peptide with Trp Gly at the *N*-terminus significantly improve its XO inhibition rate [78]. In addition, the peptide composed of simple structural amino acids connected with aromatic amino acids exhibited better inhibitory activity than the others did.

The specificity of the protease cleavage site has led to the discovery that the activity is linked to the composition of amino acids and the structure of the peptide. Therefore, enzymatic cleavage using targeted cleavage techniques to obtain peptides with the intended activity is also an attractive future research direction. Moreover, considering the need for adequate oral bioavailability and bioactivity of peptides, future prospects for marine peptide research should focus on developing separation and purification techniques with higher selectivity and resolution than conventional methods. In addition, the development of embedding techniques such as nanoemulsions and nanoliposomes would be extremely useful to identify and obtain more novel peptides at higher yields and lower costs.

### 3.4. Others

It is worth noting that seaweed contains a high proportion of secondary metabolites such as polyphenols, which are a good source of lipid-lowering bioactive substances [79]. The highest proportions of phenolic compounds in green and red algae are bromophenol, phenolic acids, and flavonoids. Phenyltannins are a composite polymer of phloroglucinol (1,3,5-trihydroxybenzene), which is the main secondary metabolite of polyphenols found only in marine brown algae. Polyphenol extracts of brown algae can activate AMPK signal transduction, thereby reducing lipid accumulation in the organism [79,80,81]. Fourteen compounds were isolated from the fermentation broth of *Streptomyces nitrospororus* YBH10-5 in the Arctic, and compound 12, farnesyl, significantly increased the expression of key proteins in Hep-G2 cells (PPAR α) and their downstream genes (*CPT-1*), acyl CoA oxidase 1 (*ACOX*), malonyl CoA decarboxylase 1 (*MCD1*), and the expression level of cholesterol 7 α hydroxylase (CYP7A1) [82]. In addition, studies have shown that naphthoquinone pigments from sea urchins also have cholesterol-lowering properties [83].

Marine fish and shellfish species also contain a substance called taurine, which is a sulfur-containing nonessential amino acid that is likely widely involved in the metabolism of living organisms, especially regulation of abnormal lipid metabolism [84]. Animal and in vitro experiments have shown that taurine supplementation significantly reduces the level of blood lipid (such as TG, TC, LDL, and HDL) [85,86]. In addition, clinical and epidemiological studies have found that taurine inhibits the process of HLP and atherosclerosis caused by HFD [86,87]. As a research hotspot, astaxanthin not only has significant antioxidant effects, but its role in regulating metabolic syndrome cannot be ignored. As early as in 2010, a clinical study demonstrated the lipid-lowering effect of astaxanthin in subjects, for the first time [88]. The results showed that astaxanthin reduce TG levels in patient serum, while HDL-C and serum adiponectin levels were significantly increase [88].

The oceans are rich in lipid-lowering bioactive substances, but most are obtained as extracted mixtures. The method for mixture extraction is simple but not conducive to an in-depth discussion of the conformational relationship of the active substances. Recently, scientists have used spectroscopy, chromatography-MS, energy spectrometry, and other techniques for purification and structural characterization in studying the molecular structure of the compounds contained in active substances [61]. These techniques have also been used to elucidate the relationship between the chemical bonding and functional groups and the activity.

## 4. Mechanisms of Marine-Derived Hypolipidemic Active Substances

Lipid metabolism is a complex process, and lipid levels in the body are related to lipid metabolism, cellular oxidative damage, and gut microbes. Lipid levels are also regulated by a variety of cholesterol synthesizing components and fat synthesis-related factors. The accumulation of lipids in blood vessels affects energy metabolism and material exchange of the surrounding tissue cells. Furthermore, disorders of these functions leads to the dormancy and decay of vascular tissue cells, leading to an inflammatory response. Therefore, in addition to monitoring LDL-C levels, inflammatory predictors represented by ultrasensitive *C*-reactive protein (hs-CRP) have been recognized as one of the factors contributing to CVD [89,90]. Moreover, LDL-C-based lipid particles entering the subendothelium of the vessel wall are constantly oxidized and modified, through a process in which reactive oxygen species (ROS) play an important role [91]. In addition, a level of ROS exceeding the physiological threshold causes lipid peroxidation [91].

### 4.1. Inhibition of Cholesterol, TG, and Fatty Acid Pathways

As shown in Figure 4, lipid metabolism consists of multiple processes, and most of the current research on lipid-lowering mechanisms still focuses on pathways of cholesterol uptake, synthesis, transport and efflux. Natural bioactive substances have been found to lower lipid levels through different pathways, such as krill oil supplements that increase the fecal output of cholesterol and bile acids, thereby stimulating cholesterol excretion by promoting bile excretion [46]. Furthermore, CPP has been shown to increase the secretion of bile acids, and thereby reduce cholesterol in the body and polyphenol extract of brown algae [80] were shown to activate AMPK. The most abundant sterol in seaweed, fucoidan, has been shown to inhibit intestinal absorption of cholesterol. Hoang et al. found that in THP-1-derived macrophages, it induced transcriptional activation of ABCA1, ABCG1 and ApoE key genes for reverse cholesterol transport, which significantly increased the efflux of cholesterol [92]. Wan et al. [42] also found that polysaccharides extracted from the green microalga *Chlorella pyrenoidosa* have hypolipidemic activity, and they further explored the molecular mechanism. Their results showed that this type of polysaccharide activates AMPK, inhibiting its downstream genes to further inhibit the synthesis of fatty acids [80]. This process controls glucolipid metabolism of the cellular energy regulator and when AMPK is activated it can regulate cholesterol synthesis by inhibiting the phosphorylation of HMG-CoA and the binding of SREBP-1c to ACC downstream genes.

### 4.2. Inhibition of Oxidative Damage Pathways

The development of HLP downregulates nuclear factor erythroid 2-related factor 2 (Nrf-2) and reduces the activity of antioxidant enzymes, which, in turn leads to oxidative stress [21,93]. Furthermore, the combination of EPA and DHA positively modulates oxidative stress and other cognitive deficits induced by HLP [21,93]. Oxidative stress plays a crucial role in the pathogenesis and progression of CVDs, and abalone viscera polysaccharides have been found to increase serum/liver SOD activity and decrease MDA in HFD mice [35,94]. Disturbances in the antioxidant system can further lead to the development of fatty liver, which manifests as an increase in oxygen radical products or a decrease in free radical scavenging enzyme activities. Increased activity of antioxidant enzymes may inhibit oxidative damage by detoxifying ROS and preventing lipid peroxidation, thereby reducing HLP [95,96].

### 4.3. Inhibition of Inflammatory Factor Pathways

Regueiras et al. [97] found that an extract of *Chlorella vulgaris* and *Chlorococcum amblystomatis* alleviated lipid accumulation in zebrafish larvae and Hep-G2 cells, and exhibited anti-inflammatory effects. Previously, HLP was believed to have no direct relationship to inflammation, which in HLP was thought to usually be caused by cellular or tissue damage [98]. However, recent data have shown that lipid disorders are closely related to the inflammatory process [99,100]. AMPK signaling inhibits the inflammatory response induced by NFκB, which regulates the expression of inflammatory genes. Accumulation of FFA activates NFκB, which further increases TNFα [101]. Interleukin-6 (IL6) and TNFα are important pro-inflammatory cytokines in HLP-associated CVDs, and are associated with plasma levels of secreted lipids [89,102,103]. In contrast, Dietary DHA supplementation activates AMPK signaling and reduces TNFα and NFκB levels, which, in turn alleviates ER stress, lipid accumulation, and inflammatory responses [21].

### 4.4. Improvement of Gut Microbial Pathways

**Figure 6:**
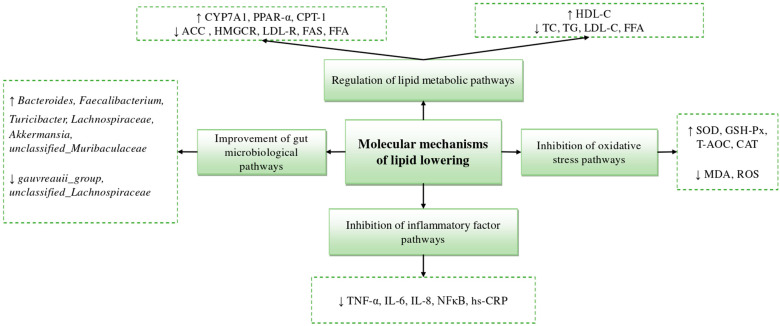
Molecular mechanisms of lipid lowering effects. ↑: Elevated levels or upward adjustments. ↓: Decrease in level or downward adjustment.

Gut flora is involved in regulating nutrient absorption and energy balance, and the composition of gut microbes varies between individuals including those with similar dietary habits, and is correlated with age, genetic, and environmental factors [104]. Several studies have shown that gut flora dysbiosis is involved in the pathologic process of CVD, including atherosclerosis, hypertension, platelet overactivity, abnormal lipid metabolism, and vascular dysfunction [105]. Most bacteria in the human and mouse gut microbiome are in the phylum, such as in the thick-walled Firmicutes and Bacteroidetes. In addition, HLP contributes to the development of chronic diseases in the host by altering the composition of the gut microbiota, leading to symptoms such as dyslipidemia, and affecting lipid metabolism [106]. Polysaccharides are rich in dietary fiber, which is beneficial to gut health, and carrageenan from *Chondrus ocellatus Holmes*., fucoidan from *Undaria pinnatifida* Suringar, and fucoidan oligosaccharides from sea cucumber (*I. badionotus* and *P. graeffei*) have been found to improve the intestinal environment of HFD mice by decreasing the abundance of Firmicutes species and increasing the abundance of Bacteroidetes [107,108]. Figure 6 shows some currently identified and characterized molecular mechanisms underlying the lipid-lowering activities of some marine-derived substances.

**Table 1:** Evaluation modeling, dosage and results of marine-derived bioactive compounds. ↑: Elevated levels or upward adjustments. ↓: Decrease in level or downward adjustment.

Category	Source	Model	Dose	Results	Reference
Marine-derived polysaccharide	Sea cucumber	Rats	40 mg/kg	TC↓, TG↓, HDL-C↑, LDL-C↓	[40]
	the chitosan of crab shells	Hamsters	5% (oral gavage)	TC↓, TG↓, HDL-C↑, Non-HDL-C↓	[41]
	*Chlorella pyrenoidosa*	Wistar rats	150 mg/kg and 300 mg/kg (oral gavage)	TC↓, TG↓, HDL-C↑, LDL-C↓	[42]
	*Sargassum pallidum*	Mice	50, 100, and 200 mg/kg (oral gavage)	TC↓, TG↓	[43]
	*Apostichopus japonicus*	Wistar strain rats	200, 400 and 800 mg/kg (oral gavage)	TC↓, TG↓,	[39]
	*Haliotis discus hannai*	Mice	200, 400 and 800 mg/kg (oral gavage)	TC↓, TG↓, HDL-C↑, LDL-C↓, MDA↓	[35]
	*Isostichopus badionotus*; *Pearsonothuria graeffei*	C57BL/6J mice	80 mg/kg (oral gavage)	-	[108]
Marine-derived unsaturated fatty acid	Fish oil	Zebrafish	diet containing 20% (*w*/*w*)	Lipid accumulation ↓	[56]
	sea cucumber	C57BL/6 mice	0.3% (oral gavage)	TC↓, TG↓, NEFA↓	[22]
	Krill oil	Sprague Dawley (SD) rats	100 mg/kg, 200 mg/kg (oral gavage)	TC↓, TG↓, HDL-C↑, LDL-C↓	[59]
	Krill oil	AopE−/− mice	1.5 mg/kg (oral gavage)	TC↓, TG↓, HDL-C↑, LDL-C↓	[60]
Marine-derived bioactive peptide	salmon bone frames	fa/fa Zucker rats; fa/fa Zucker rats	264.9 mg/kg; 233.9 mg/kg (oral gavage)	TC↓, HDL-C↑,	[73]
	*Sphyrna mokarran* skin	Wistar strain albino rats	600 mg/kg (oral gavage)	TC↓, TG↓, HDL-C↑, LDL-C↓, VLDL-C↓	[73]
	*Tuna skin*	ICR mice	300 mg/kg (oral gavage)	TC↓, TG↓, HDL-C↑, LDL-C↓	[64]
	*Raja kenojei*	C57BL6/J mice	100 mg/kg (oral gavage)	TG↓, NEFA↓, HDL-C↑, LDL-C↓	[23]
	*Zosterissessor ophiocephalus*	Rats	400 mg/kg (oral gavage)	TC↓, TG↓, LDL-C↓	[46]
	*Miichthys miiuy*	HepG2	50, 100 μM	TC↓, TG↓	[76]

## 5. Discussion

Marine bioactive substances are characterised by a wide range of sources and complex structures, which makes them particularly useful resources for the development of products with lipid-lowering effect. Current research studies on marine-derived lipid-lowering bioactive substances are increasing both in China and globally, but some problems, including the following, still need to be comprehensively addressed in relevant research studies. (1) Research on the specific composition, structural characterization, specific constitutive relationship, and molecular mechanism of active substances is not robust enough. Furthermore, there are few reports on the effects of active substances on metabolic processes of organisms. (2) The components of marine-derived bioactive substances are diverse and complex, and there are still difficulties associated with processes for screening out bioactive molecules. (3) There are few studies on the transfer and absorption process of the identified bioactive substances in the body, and their specific extent of their bioavailability is unclear. (4) Consider that cholesterol and bile acid metabolism in test animals is different from that of humans. Lipoprotein metabolism in rodents is primarily based on HDL, whereas in humans it is based on LDL. Therefore, experimental results in mice and rats may not automatically translate to humans. (5) Except for some fish oil preparations, marine-derived bioactive substances have not yet been widely used in clinical trials or established on the market. However, the development of computer technology, virtual screening, molecular docking, and other technologies accompanied by high-throughput sequencing technology is also advancing and flourishing, which has far-reaching significance to the future development of a variety of marine-based lipid-lowering bioactive substances. Furthermore, these developments have an impact on in-depth discussions of the molecular mechanism underlying the activity of these substances, including the following. (1) The use of clearly defined structures of bioactive substances can be beneficial to conducting high-volume virtual screening using computer platforms, and further molecular docking using clear complexes. (2) Using transcriptomics or metabolomics to study the specific pathways of bioactive substances mediating the blood lipid lowering effects and identifying more targets. (3) Clarifying the extent of the transportation and absorption of bioactive substances in the body, would contribute to maximizing their bioavailability using auxiliary means. (4) More stable bioactive substances can be verified in clinical trials after in vitro and in vivo testing in experimental animals. (5) Due to climatic and environmental changes in recent years, the question of how to obtain active ingredients from limited marine resources on a long term basis is one that deserves some thought, and perhaps synthetic techniques could also be considered.

## References

[1] Muhammad J., Garrison S., Rachel N., Deliana K., Patricia R. (2022). The cost-effectiveness of hyperlipidemia medication in low- and middle-income countries: A review. Glob. Heart.

[2] Lee N., Tom B., Dawit Z. (2023). Global Trends in Atherosclerotic Cardiovascular Disease. Clin. Ther..

[3] Hung P.M., Thanh V.H., Van Sy H., Duc D.Q., Tuan V.A., Tran A.T., Brizuela G.E., Tran H.B. (2022). Adherence to hypertension and dyslipidemia treatment and its implication on control of cardiovascular disease in Vietnam: A semi-systematic review. Medicine.

[4] Attardo S., Musumeci O., Velardo D., Toscano A. (2022). Statins neuromuscular adverse effects. Int. J. Mol. Sci..

[5] Gabriela P., Andreea F., Anca B., Claudiu M., Anca M., Manuela P., Minodora T., Felicia G. (2022). Post-Marketing surveillance of statins—A descriptive analysis of psychiatric adverse reactions in EudraVigilance. Pharmaceuticals.

[6] Manue U., Tatiana P., Carol P., Miguel U. (2021). Statin associated adverse reactions in Latin America: A scoping review. BMJ. Open.

[7] Thompson F.E., Subar A.F., Coulston A.M. (2005). Dietary Assessment Methodology. Nutrition in the Prevention and Treatment of Disease.

[8] Thompson C., Kruger H., Thompson F. (2017). Unlocking marine biotechnology in the developing world. Trends Biotechnol..

[9] Florean C., Dicato M., Diederich M. (2020). Immune-modulating and anti-inflammatory marine compounds against cancer. Semin. Cancer Biol..

[10] Lobine D., Rengasamy K., Mahomoodally F. (2022). Functional foods and bioactive ingredients harnessed from the ocean: Current status and future perspectives. Crit. Rev. Food Sci. Nutr..

[11] Correia-da-Silva M., Sousa E., Pinto M. (2017). Anticancer and cancer preventive compounds from edible marine organisms. Semin. Cancer Biol..

[12] Yang J., Zhang T., Yu Z., Wang C., Zhao Y., Wang Y., Xue C. (2022). Taurine alleviates trimethylamine N-Oxide-Induced atherosclerosis by regulating bile acid metabolism in ApoE(-/-) mice. J. Agric. Food Chem..

[13] Yang Y., Woo J., Seo Y., Lee K., Lim Y., Choi J. (2016). Protective effect of brown alga phlorotannins against hyper-inflammatory responses in lipopolysaccharide-induced sepsis models. J. Agric. Food Chem..

[14] Kalita P., Ahmed A., Sen S., Chakraborty R. (2022). A comprehensive review on polysaccharides with hypolipidemic activity: Occurrence, chemistry and molecular mechanism. Int. J. Biol. Macromol..

[15] Rodriguez-Garcia M., Alcaide P. (2021). Vascular Inflammation and hyperlipidemia: The neutrophil within. JACC Basic Transl. Sci..

[16] Song D., Jiang J. (2017). Hypolipidemic components from medicine food homology species used in china: Pharmacological and health effects. Arch. Med. Res..

[17] Salyamova A., Khromova A., Kvasova O., Burko N., Oleinikov V. (2021). The incidence of muscle tissue damage during therapy with atorvastatin in patients after STEMI. Eur. Heart J..

[18] Carlson L. (2010). Nicotinic acid: The broad-spectrum lipid drug. A 50th anniversary review. J. Intern. Med..

[19] Durham S., Covington E., Clemmons K. (2018). Hepatotoxicity upon using niacin to pass a drug test: A case report. J. Am. Pharm. Assoc..

[20] Vavlukis M., Vavlukis A. (2018). Adding ezetimibe to statin therapy: Latest evidence and clinical implications. Drugs Context.

[21] Huang X., Sun J., Bian C., Ji S., Ji H. (2022). Docosahexaenoic acid lessens hepatic lipid accumulation and inflammation via the AMP-activated protein kinase and endoplasmic reticulum stress signaling pathways in grass carp (*Ctenopharyngodon idella*). Food Funct..

[22] Tian Y., Liu Y., Xue C., Wang J., Wang Y., Xu J., Li Z. (2020). Exogenous natural EPA-enriched phosphatidylcholine and phosphatidylethanolamine ameliorate lipid accumulation and insulin resistance via activation of PPARα/γ in mice. Food Funct..

[23] Woo J., Song Y., Kang K., Noh J. (2018). Anti-Obesity effects of collagen peptide derived from skate (*Raja kenojei*) skin through regulation of lipid metabolism. Mar. Drugs.

[24] Pozharitskaya O.N., Shikov A.N., Laakso I., Seppänen-Laakso T., Makarenko I.E., Faustova N.M., Makarova M.N., Makarov V.G. (2015). Bioactivity and chemical characterization of gonads of green sea urchin *Strongylocentrotus droebachiensis* from Barents Sea. J. Funct. Foods.

[25] Pavlicevic M., Maestri E., Marmiroli M. (2020). Marine Bioactive Peptides-An Overview of Generation, Structure and Application with a Focus on Food Sources. Mar. Drugs.

[26] Lomartire S., Gonçalves A.M. (2022). An Overview of Potential Seaweed-Derived Bioactive Compounds for Pharmaceutical Applications. Mar. Drugs.

[27] Pozharitskaya O.N., Obluchinskaya E.D., Shikov A.N. (2020). Mechanisms of Bioactivities of Fucoidan from the Brown Seaweed *Fucus vesiculosus* L. of the Barents Sea. Mar. Drugs.

[28] Kim M., Yang I., Lee H., Lee J., Kim K. (2020). Lipid-modifying effects of krill oil vs fish oil: A network meta-analysis. Nutr. Rev..

[29] Wang Z., Xu Z., Yang X., Li M., Yip R., Li Y., Chen H. (2023). Current application and modification strategy of marine polysaccharides in tissue regeneration: A review. Biomater. Adv..

[30] Arnosti C., Wietz M., Brinkhoff T., Hehemann J., Probandt D., Zeugner L., Amann R. (2021). The biogeochemistry of marine polysaccharides: Sources, inventories, and bacterial drivers of the carbohydrate cycle. Annu. Rev. Mar. Sci..

[31] Perumal P., Dong C., Chauhan A., Anisha G., Kadri M., Chen C., Singhania R., Patel A. (2023). Advances in oligosaccharides production from algal sources and potential applications. Biotechnol. Adv..

[32] Liu Z., Ai C., Lin X., Guo X., Song S., Zhu B. (2023). Sea cucumber sulfated polysaccharides and *Lactobacillus gasseri* synergistically ameliorate the overweight induced by altered gut microbiota in mice. Food Funct..

[33] Qu H., Wu Y., Luo Z., Dai C. (2023). An efficient approach for extraction of polysaccharide from abalone (Haliotis Discus Hannai Ino) viscera by natural deep eutectic solvent. Int. J. Biol. Macromol..

[34] Sun J., Song S., Ai C., Zhu B. (2022). A sulfated abalone polysaccharide inhibited SARS-CoV-2 infection of Vero E6 Cells in vitro. Foods.

[35] Liu B., Jia Z., Li C., Chen J., Fang T. (2020). Hypolipidemic and anti-atherogenic activities of crude polysaccharides from abalone viscera. Food Sci. Nutr..

[36] Rajesh J., Ahmad A., Daneshwar P., Nadeem N., Abdulwahed F., Zhang Y. (2023). Marine Microbial Polysaccharides: An untapped resource for biotechnological applications. Mar. Drugs.

[37] Yan M., Mao M., Liu X., Wang S., Xia Z., Cao S., Li J., Qin L., Xian H. (2016). Extracellular polysaccharide with novel structure and antioxidant property produced by the deep-sea fungus *Aspergillus versicolor* N(2)bC. Carbohydr. Polym..

[38] Qiu J., Shi W., Miao J., Hu H., Gao Y. (2023). Extraction, isolation, screening, and preliminary characterization of polysaccharides with anti-oxidant activities from *Oudemansiella raphanipies*. Polymers.

[39] Liu X., Sun Z., Zhang M., Meng X., Xia X., Yuan W., Xue F., Liu C. (2012). Antioxidant and antihyperlipidemic activities of polysaccharides from sea cucumber *Apostichopus japonicus*. Carbohydr. Polym..

[40] Liu X., Sun Z., Zhang M., Meng X., Xia X., Yuan W., Xue F., Liu C. (2017). Macromolecular properties and hypolipidemic effects of four sulfated polysaccharides from sea cucumbers. Carbohydr. Polym..

[41] Abdo A., Zhang C., Al-Dalali S., Hou Y., Gao J., Yahya M., Saleh A., Aleryani H., Al-Zamani Z., Sang Y. (2023). Marine chitosan-oligosaccharide ameliorated plasma cholesterol in hypercholesterolemic hamsters by modifying the gut microflora, bile acids, and short-chain fatty acids. Nutrients.

[42] Wan X., Ai C., Chen Y., Gao X., Zhong R., Liu B., Chen X., Zhao C. (2020). Physicochemical characterization of a polysaccharide from green microalga chlorella pyrenoidosa and its hypolipidemic activity via gut microbiota regulation in rats. J. Agric. Food Chem..

[43] Yuan D., Huang Q., Li C., Fu X. (2022). A polysaccharide from Sargassum pallidum reduces obesity in high-fat diet-induced obese mice by modulating glycolipid metabolism. Food Funct..

[44] Wang J., Wu C., Yan C., Chen H., You S., Sheng S., Wu F., Wang J. (2015). Silkworm pupa oil exerts hypolipidemic and antioxidative effects on rat model of high-fat diet induced hyperlipidemia. FASEB J..

[45] Oršolić N., Jurčević N., Đikić D., Rogić D., Odeh D., Balta V., Junaković E., Terzić S., Jutrić D. (2019). Effect of propolis on diet-induced hyperlipidemia and atherogenic indices in mice. Antioxidants.

[46] Kim O., Yun J., Kim D., Park S., Lee C., Go E., Kim J., Park S., Lee J. (2022). Krill oil inhibits cholesterol synthesis and stimulated cholesterol excretion in hypercholesterolemic rats. Mar. Drugs.

[47] Zhang T., Xu J., Wang Y., Xue C. (2019). Health benefits of dietary marine DHA/EPA-enriched glycerophospholipids. Prog. Lipid Res..

[48] Yang S. (2021). A New Perspective on Fish Oil: The prevention of alcoholic liver disease: Review. J. Oleo Sci..

[49] Liu Y., Li Q., Wang H., Zhao X., Li N., Zhang H., Chen G., Liu Z. (2019). Fish oil alleviates circadian bile composition dysregulation in male mice with NAFLD. J. Nutr. Biochem..

[50] Calder P. (2021). Beneficial Outcomes of omega-6 and omega-3 polyunsaturated fatty aacids on human health: An update for 2021. Nutrients.

[51] Kimura R., Takahashi N., Lin S., Goto T., Murota K., Nakata R., Inoue H., Kawada T. (2013). DHA attenuates postprandial hyperlipidemia via activating PPARα in intestinal epithelial cells. J. Lipid Res..

[52] Chong S.Y., Wang X., van Bloois L., Huang C., Syeda N.S., Zhang S., Ting H.J., Nair V., Lin Y., Lou C.K.L. (2023). Injectable liposomal docosahexaenoic acid alleviates atherosclerosis progression and enhances plaque stability. J. Control Release.

[53] Skulas-Ray A.C., Wilson P.W., Harris W.S., Brinton E.A., Kris-Etherton P.M., Richter C.K., Jacobson T.A., Engler M.B., Miller M., Robinson J.G. (2019). Omega-3 fatty acids for the management of hypertriglyceridemia: A science advisory from the American Heart Association. Circulation.

[54] Grundy S.M., Stone N.J., Bailey A.L., Beam C., Birtcher K.K., Blumenthal R.S., Braun L.T., de Ferranti S., Faiella-Tommasino J., Forman D.E. (2019). 2018 AHA/ACC/AACVPR/AAPA/ABC/ACPM/ADA/AGS/APhA/ASPC/NLA/PCNA Guideline on the management of blood cholesterol: A report of the American College of Cardiology/American Heart Association task force on clinical practice guideline. Circulation.

[55] François M., Colin B., Catapano A., Koskinas K.C., Casula M., Badimon L., Chapman M.J., De Backer G.G., Delgado V., Ference B.A. (2020). ESC/EAS Guidelines for the management of dyslipidaemias: Lipid modification to reduce cardiovascular risk. Eur. Heart J..

[56] Sabarinathan S., Rani R. (2022). Protective effect of fish oil targeting hadhaa (3-ketoacyl-CoA thiolase, alpha subunit a and hadhb (3-ketoacyl-CoA thiolase, beta subunit) against high cholesterol diet (HCD) induced atherosclerosis on Zebrafish. Obes. Med..

[57] Hwang S., Kim Y., Kim J., Chun Y., Kwon Y., Ku S., Song C. (2022). Preventive and therapeutic effects of krill oil on obesity and obesity-induced metabolic syndromes in high-fat diet-fed mice. Mar. Drugs.

[58] Stine M., Kirsten B. (2015). Comparison of bioavailability of krill oil versus fish oil and health effect. Vasc. Health Risk Manag..

[59] Guo P., Xue M., Teng X., Wang Y., Ren R., Han J., Zhang H., Tian Y., Liang H. (2022). Antarctic krill oil ameliorates liver injury in rats exposed to alcohol by regulating bile acids metabolism and gut microbiota. J. Nutr. Biochem..

[60] Liang X., Zhang Z., Lv Y., Lu H., Liu T., Yi H., Zhao M., Zhang L., Gong P. (2021). Krill oil combined with *Bifidobacterium animalis* subsp. lactis F1-7 alleviates the atherosclerosis of ApoE−/− mice. Foods.

[61] Hosseini S.F., Rezaei M., Mcclements D.J. (2022). Bioactive functional ingredients from aquatic origin: A review of recent progress in marine-derived nutraceuticals. Crit. Rev. Food Sci. Nutr..

[62] Rodriguez D., Lavie C.J., Elagizi A., Milani R.V. (2022). Update on Omega-3 Polyunsaturated Fatty Acids on Cardiovascular Health. Nutrients.

[63] Sara K., Zahra F., Mahdi J.S. (2020). A systematic review and meta-analysis of fish oil encapsulation within different micro/nanocarriers. Crit. Rev. Food Sci. Nutr..

[64] Nirmal N.P., Rajput M.S., Rathod N.B., Mudgil P., Pati S., Bono G., Nalinanon S., Li L., Maqsood S. (2022). Structural characteristic and molecular docking simulation of fish protein-derived peptides: Recent updates on antioxidant, anti-hypertensive and anti-diabetic peptides. Food Chem..

[65] Zhang M., Liu S., Yang X., Zhao X., Wang C., Xu H. (2021). Immunomodulatory effects of different molecular weight *sporisorium reilianum* polypeptides on LPS-induced RAW264.7 macrophages. Food Biosci..

[66] Ma J., Zeng X., Zhou M., Cheng L., Ren D. (2021). Inhibitory effect of low-molecular-weight peptides (0–3kDa) from Spirulina platensis on H_2_O_2_-induced oxidative damage in L02 human liver cells. Bioresour. Bioprocess.

[67] Liu X., Zhang M., Zhang C., Liu C. (2012). Angiotensin converting enzyme (ACE) inhibitory, antihypertensive and antihyperlipidaemic activities of protein hydrolysates from *Rhopilema esculentum*. Food Chem..

[68] Lin Y., Tsai J., Hung L., Pan B. (2011). Plasma lipid regulatory effect of compounded freshwater clam hydrolysate and *Gracilaria* insoluble dietary fibre. Food Chem..

[69] Khaled H., Ghlissi Z., Chtourou Y., Hakim A., Ktari N., Fatma M., Barkia A., Sahnoun Z., Nasri M. (2012). Effect of protein hydrolysates from sardinelle (*Sardinella aurita*) on the oxidative status and blood lipid profile of cholesterol-fed rats. Food Res. Int..

[70] Mantecón L., Moyano R., Cameán A.M., Jos A. (2019). Safety assessment of a lyophilized biomass of *Tetraselmis chuii* (TetraSOD) in a 90 day feeding study. FCT.

[71] Gil-Cardoso K., Josep M., Caimari A., Lama C., Torres S., Mantecón L., Infante C. (2022). TetraSOD^®^, a unique marine microalgae ingredient, promotes an antioxidant and anti-Inflammatory status in a metabolic syndrome-Induced model in rats. Nutrients.

[72] Divya K., Raman S., Dara P., Jacob R., Mathew S., Rangasamy A., Nagarajarao R. (2022). In vivo anti-lipidemic and antioxidant potential of collagen peptides obtained from great hammerhead shark skin waste. J. Food. Sci. Technol..

[73] Wergedahl H., Liaset B., Gudbrandsen O., Lied E., Espe M., Muna Z., Mørk S., Berge R. (2004). Fish protein hydrolysate reduces plasma total cholesterol, increases the proportion of HDL cholesterol, and lowers acyl-CoA:cholesterol acyltransferase activity in liver of Zucker rats. J. Nutr..

[74] Lee E., Hur J., Ham S., Jo Y., Lee S., Choi M., Seo H. (2017). Fish collagen peptide inhibits the adipogenic differentiation of preadipocytes and ameliorates obesity in high fat diet-fed mice. Int. J. Biol. Macromol..

[75] Schmidt C.A., Wilson D.T., Cooke I., Potriquet J., Tungatt K., Muruganandah V., Boote C., Kuek F., Miles J.J., Kupz A. (2020). Identification and Characterization of a Peptide from the Stony Coral *Heliofungia actiniformis*. J. Nat. Prod..

[76] Wang Y., Pan X., He Y., Chi C., Wang B. (2020). Hypolipidemic activities of two pentapeptides (VIAPW and IRWWW) from Miiuy Croaker (*Miichthys miiuy*) muscle on lipid accumulation in HepG2 Cells through regulation of AMPK pathway. Appl. Sci..

[77] Agu P.C., Afiukwa C.A., Orji O.U. (2023). Molecular docking as a tool for the discovery of molecular targets of nutraceuticals in diseases management. Sci. Rep..

[78] Zhao Q., Jiang X., Mao Z., Zhang J., Sun J., Mao X. (2023). Exploration, sequence optimization and mechanism analysis of novel xanthine oxidase inhibitory peptide from *Ostrea rivularis* Gould. Food Chem..

[79] Margaret M., Aimee L., Maxine P., Lisa R. (2018). Do marine algal polyphenols have antidiabetic, antihyperlipidemic or anti-inflammatory effects in humans? A systematic review. Crit. Rev. Food. Sci..

[80] Manuel G., Alba R., Francesca A., Julio G. (2018). Potential role of seaweed polyphenols in cardiovascular-associated disorders. Mar. Drugs.

[81] Choi H., Jeon H., Le O., Lee B. (2015). Dieckol, a major phlorotannin in Ecklonia cava, suppresses lipid accumulation in the adipocytes of high-fat diet-fed zebrafish and mice: Inhibition of early adipogenesis via cell-cycle arrest and AMPKα activation. Mol. Nutr. Food Res..

[82] Wells M., Potin P., Craigie J., Raven J.A., Merchant S.S., Helliwell K.E., Smith A.G., Camire M.E., Brawley S.H. (2017). Algae as nutritional and functional food sources: Revisiting our understanding. J. Appl. Phycol..

[83] Shikov A.N., Pozharitskaya O.N., Krishtopina A.S. (2018). Naphthoquinone pigments from sea urchins: Chemistry and pharmacology. Phytochem. Rev..

[84] Liu D., Yang A., Wu C., Guo G., Proksch P., Lin W. (2014). Lipid-lowering effects of farnesylquinone and related analogues from the marine-derived *Streptomyces nitrosporeus*. Bioorg. Med. Chem. Lett..

[85] Abebe W., Mozaffari M. (2011). Role of taurine in the vasculature: An overview of experimental and human studies. Am. J. Cardiovasc. Dis..

[86] Dong Y., Li X., Liu Y., Gao J., Tao J. (2021). The molecular targets of taurine confer anti-hyperlipidemic effects. Life Sci..

[87] Kim K., Oh D., Kim J., Lee B.G., You J.S., Chang K.J., Chung H., Yoo M.C., Yang H.I., Kang J.H. (2012). Taurine ameliorates hyperglycemia and dyslipidemia by reducing insulin resistance and leptin level in Otsuka Long-Evans Tokushima fatty (OLETF) rats with long-term diabetes. Exp. Mol. Med..

[88] Yoshida H., Yanai H., Ito K., Tomono Y., Koikeda T., Tsukahara H., Tada N. (2010). Administration of natural astaxanthin increases serum HDL-cholesterol and adiponectin in subjects with mild hyperlipidemia. Atherosclerosis.

[89] Yang L., Yue Q., Fang F., Zhang Y., Liu P., Zhang Z., Wang G., Chen S., Wu S., Yang X. (2023). Effect of dual residual risk of cholesterol and inflammation on all-cause mortality in patients with cardiovascular disease. Cardiovasc. Diabetol..

[90] Burger P., Koudstaal S., Mosterd A., Fiolet A.T., Teraa M., van der Meer M.G., Cramer M.J., Visseren F.L., Ridker P.M., Dorresteijn J.A. (2023). C-Reactive protein and risk of incident heart failure in patients with cardiovascular disease. J. Am. Coll. Cardiol..

[91] Münzel T., Gori T., Bruno R. (2010). Is oxidative stress a therapeutic target in cardiovascular disease?. Eur. Heart J..

[92] Hoang M.H., Jia Y., Jun H.J., Lee J.H., Lee B.Y., Lee S.J. (2012). Fucosterol is a selective liver X receptor modulator that regulates the expression of key genes in cholesterol homeostasis in macrophages, hepatocytes, and intestinal cells. J. Agric. Food Chem..

[93] Uppin V., Acharya P., Kempaiah B., Talahalli R. (2020). Zerumbone augments cognitive enhancement potentials of EPA+DHA: Insight from a hyperlipidaemic rat model. Br. J. Nutr..

[94] Liu Y., Ma N., Sun X., Duan M., Luo T., Jiang P., Jiang G., Song S., Ai C. (2019). Effect of intake pattern of sulfated polysaccharides on its biological activity in high fat diet-fed mice. Int. J. Biol. Macromol..

[95] Jia R., Li Z., Wu J., Ou Z., Zhu Q., Sun B., Lin L., Zhao M. (2020). Physicochemical properties of polysaccharide fractions from *Sargassum fusiforme* and their hypoglycemic and hypolipidemic activities in type 2 diabetic rats. Int. J. Biol. Macromol..

[96] Li H., Zhang M., Ma G. (2010). Hypolipidemic effect of the polysaccharide from *Pholiota nameko*. Nutrition.

[97] Urbatzka R. (2021). Potential anti-obesity, anti-steatosis, and anti-inflammatory properties of extracts from the microalgae chlorella vulgaris and chlorococcum amblystomatis under different growth conditions. Mar. Drugs.

[98] Tietge U. (2014). Hyperlipidemia and cardiovascular disease: Inflammation, dyslipidemia, and atherosclerosis. Curr. Opin. Lipidol..

[99] Hofmaenner D., Kleyman A., Press A., Bauer M., Singe M. (2022). The many roles of cholesterol in sepsis: A review. Am. J. Respir. Crit. Care Med..

[100] Libby P., Buring J., Badimon L., Hansson G., Deanfield J., Bittencourt M., Tokgözoğlu L., Lewis E. (2019). Atherosclerosis. Nat. Rev. Dis. Primers.

[101] Zou B., Ge Z., Zhang Y., Du J., Xua Z., Li C. (2014). Persimmon Tannin accounts for hypolipidemic effects of persimmon through activating of AMPK and suppressing NF-kB activation and inflammatory responses in High-Fat Diet Rats. Food. Funct..

[102] Siasos G., Tousoulis D., Oikonomou E., Zaromitidou M., Stefanadis C., Papavassiliou A. (2011). inflammatory markers in hyperlipidemia: From experimental models to clinical practice. Curr. Pharm. Des..

[103] Wang S., Wu D., Matthan N., Lamon-Fava S., Lecker J., Aortic A. (2010). Macrophage lipid accumulation and inflammatory response in LDL receptor Null mice fed an atherogenic diet. Lipids.

[104] Zwartjes M., Gerdes V., Nieuwdorp M. (2021). The role of gut microbiota and its produced metabolites in obesity, dyslipidemia, adipocyte dysfunction, and its interventions. Metabolites.

[105] Wang Z., Klipfell E., Bennett B., Koeth R., Levison B.S., DuGar B., Feldstein A.E., Britt E.B., Fu X., Chung Y.M. (2011). Gut flora metabolism of phosphatidylcholine promotes cardiovascular disease. Nature.

[106] Kim K., Gu W., Lee I., Joh E., Kim D. (2012). High fat diet-induced gut microbiota exacerbates inflammation and obesity in mice via the TLR4 signaling pathway. PLoS ONE.

[107] Aroa L., Jose M., Alicia D., Alexandre L., Alejandra C., Carlos M., Alberto C. (2020). Potential Use of Marine seaweeds as prebiotics: A review. Molecules.

[108] Li S., Li J., Mao G., Yan L., Hu Y., Ye X., Tian D., Linhardt R.J., Chen S. (2019). Effect of the sulfation pattern of sea cucumber-derived fucoidan oligosaccharides on modulating metabolic syndromes and gut microbiota dysbiosis caused by HFD in mice. J. Funct. Foods.

